# Supplementary material for: Measurement properties of pain scoring instruments in farm animals: A systematic review protocol using the COSMIN checklist
Source: PLoS One. 2021 May 14;16(5):e0251435. doi: 10.1371/journal.pone.0251435 (PMC8121329; doi:10.1371/journal.pone.0251435)
Supplement: Attachment — Submitted filename: Plos 1 review.docx [file pone.0251435.s002.docx]

Line 59: It is important to consider that pain management practices can be labor intensive. Thus, whatever pain management practices are used must consider labor. As we know, labor is hard to recruit and retain in agriculture.

Line 63: It is also important to consider that using pain scales in production systems that use group housing is very difficult. Identifying compromised animals in a timely fashion is difficult given that the number of employees to the number of animals is very disproportionate and thus, time consuming. Additionally, the high turnover of ag employees makes it even more difficult to identify these animals as employees must be trained to identify these animals. While pain scales work great in controlled settings, in commercial settings they don’t work all that great- due to the time constraints and number of animals per pen. Please ensure that this is mentioned in the paper.
