# Supplementary material for: Measurement properties of pain scoring instruments in farm animals: A systematic review protocol using the COSMIN checklist
Source: PLoS One. 2021 May 14;16(5):e0251435. doi: 10.1371/journal.pone.0251435 (PMC8121329; doi:10.1371/journal.pone.0251435)
Supplement: Attachment — Submitted filename: Response to reviewers.docx [file pone.0251435.s004.docx]

Manuscript PONE-D-20-37059R1

Authors: Thank you very much for your comments.

Reviewer #1: This is a well written paper. The topic is of upmost importance. Validated scales are difficult to find and use. I think this manuscript provides information that will be very helpful in the development of such scales.

Answer: Thank you for your thoughtful review.

Reviewer #2: This manuscript is greatly improved following suggested reviewers' comments and my recommendation is to accept in current form.

Answer: Thank you for your thoughtful review.

Reviewer #3: Dear authors

Thank you for the revised manuscript. I have only one comment left: I notice that 'pain behaviours' are now changed to 'pain-related behaviours'.

In the first version of the manuscript, I mentioned that I know that 'pain behaviour' it is used in sci writing, but I advised you to not do that, as it is what some people consider a weasel word, because many of the behaviours potentially indicating pain are not specific to pain, and by calling it 'pain behaviour' the author may give the reader the impression that we know that these behaviours indicate pain - i.e. it becomes kind of self-fulfilling. Check for example the writing by Nicole Nelson.

The re-wording from 'pain behaviour' to 'pain-related' behaviors is a move in the right direction, but I still consider it problematic. I would write 'behaviours potentially indicating pain' or something like that.

Answer: Thank you again for your comments. We have now replaced “pain-related behaviors” to the wording (or similar) suggested by the reviewer throughout the manuscript.
